# A novel biomarker of MMP-cleaved prolargin is elevated in patients with psoriatic arthritis

**DOI:** 10.1038/s41598-020-70327-0

**Published:** 2020-08-11

**Authors:** Dovile Sinkeviciute, Solveig Skovlund Groen, Shu Sun, Tina Manon-Jensen, Anders Aspberg, Patrik Önnerfjord, Anne-Christine Bay-Jensen, Salome Kristensen, Signe Holm Nielsen

**Affiliations:** 1 Nordic Bioscience, Biomarkers and Research, Herlev Hovedgade 205-207, Herlev, Denmark; 2 Department of Clinical Sciences Lund, Lund University, Lund, Sweden; 3 Department of Biomedical Sciences, University of Copenhagen, Copenhagen, Denmark; 4 Department of Rheumatology, Aalborg University Hospital, Aalborg, Denmark; 5 Department of Biotechnology and Biomedicine, Technical University of Denmark, Kgs. Lyngby, Denmark

**Keywords:** Biomarkers, ELISA, Psoriatic arthritis

## Abstract

Psoriatic arthritis (PsA) is a chronic musculoskeletal inflammatory disease found in up to 30% of psoriasis patients. Prolargin—an extracellular matrix (ECM) protein present in cartilage and tendon—has been previously shown elevated in serum of patients with psoriasis. ECM protein fragments can reflect tissue turnover and pathological changes; thus, this study aimed to develop, validate and characterize a novel biomarker PROM targeting a matrix metalloproteinase (MMP)-cleaved prolargin neo-epitope, and to evaluate it as a biomarker for PsA. A competitive ELISA was developed with a monoclonal mouse antibody; dilution- and spiking-recovery, inter- and intra-variation, and accuracy were evaluated. Serum levels were evaluated in 55 healthy individuals and 111 patients diagnosed with PsA by the CASPAR criteria. Results indicated that the PROM assay was specific for the neo-epitope. Inter- and intra- assay variations were 11% and 4%, respectively. PROM was elevated (p = 0.0003) in patients with PsA (median: 0.24, IQR: 0.19–0.31) compared to healthy controls (0.18; 0.14–0.23) at baseline. AUROC for separation of healthy controls from PsA patients was 0.674 (95% CI 0.597–0.744, P < 0.001). In conclusion, MMP-cleaved prolargin can be quantified in serum by the PROM assay and has the potential to separate patients with PsA from healthy controls.

## Introduction

Psoriatic arthritis (PsA) is an inflammatory chronic joint disease that is found in up to 30% of psoriasis patients and can precede the skin manifestations of the disease^1,2^. Risk factors for developing PsA are psoriasis severity, family history of the disease, psoriatic nail changes and polymorphisms in human leukocyte antigen (HLA) and major histocompatibility complex (MHC) class I polypeptide-related sequence A (MICA) loci^3^. Men and women are equally affected^4^. PsA typically affects the large joints, especially joints of the lower extremities, and distal joints of the fingers and toes, however it can also affect the spinal and sacroiliac joints of the pelvis^5^. The number of involved joints varies among patients—several or only 1–2 joint can be affected, which leads to diverse clinical features, resulting in difficulties when diagnosing patients^4^. Potential complications of PsA include eye problems, such as conjunctivitis or uveitis, cardiovascular disease, and arthritis mutilans—a severe, painful and disabling form of joint disease, where small bones of the hands are destroyed, leading to permanent deformity and disability^4,6^.

The most common symptoms of PsA are joint and tendon pain, swollen fingers and toes, and lower back pain^4^. No specific diagnostic test is available for psoriatic arthritis^4^. Instead, the diagnosis is based on a combination of clinical criteria, blood tests, including erythrocyte sedimentation rate (ESR) and C-reactive protein (CRP) to check for inflammation and x-rays or MRI scans for joint damage^3,5^. There is no cure for the PsA at the moment, and the treatment focuses on symptom relief and prevention of joint involvement^7^. Non-steroid anti-inflammatory drugs (NSAIDs), corticosteroids, disease-modifying anti-rheumatic drugs (DMARDs) and biological therapies are currently used to relieve pain, protect the joints, and maintain mobility^7^. PsA gets progressively worse without intervention, but if diagnosed and treated early the disease progression can be slowed down and structural joint damage delayed or prevented^8^. Considering that many patients with psoriasis have undiagnosed PsA, and nearly 50% of patients with PsA will develop erosions in the first 2 years of the disease, predicting arthritis prior to its onset is vital for avoiding the damage^9–12^.

Biomarkers are measurable biological indicators of disease activity that may be used to predict future disease, measure current disease activity, or quantify therapeutic efficacy. Therefore, biomarkers have been identified as a relevant research gap in PsA^13^. In rheumatic diseases, the biomarkers are usually either genetic, serological, cellular, synovial or imaging type^14,15^. Serological biomarkers obtained from peripheral blood are of particular interest since they can be easily accessible at the clinic. So far, some studies suggest serum interleukin (IL)-2, IL-10, MMP3 and vascular endothelial growth factor (VEGF) may be used to discriminate patients with PsA from patients with psoriasis^14^. Collagen fragments, such as a released N-terminal pro-peptide of type II collagen *(*PRO-C2) have been shown to be increased in patients with PsA compared to healthy controls, thus potentially allowing for screening of the disease^16^.

Prolargin, also known as PRELP (proline/arginine-rich end leucine-rich repeat protein) is a 58 kDa proteoglycan and a member of the small leucine-rich proteoglycan (SLRP) family^17^. It is found in variety of extracellular matrices, including cartilage matrix and basement membranes^18,19^. It is also found in tissues such as in the sclera, kidney, tendon, skin, liver, lung, and heart^18,20^. Prolargin has been shown to inhibit all three pathways of complement system and has been suggested to have a role in joint disease^21^. In PsA patients, serum complement component 3 (C3) levels were reported higher than in control group^22^. Since prolargin has previously been shown to inhibit the alternative pathway C3 convertase^21^, and its expression to be 1.35-fold higher in psoriasis group compared to controls in miR-31 microarray assay^23^, it is likely relevant to PsA as well. Indeed, prolargin was recently suggested as a new protein candidate indicative of response to anti-tumor necrosis factor (TNF)-α in PsA, thus warranting further investigation of this protein in this disease^24^.

In mass spectrometry analysis of matrix metalloproteinase (MMP)-degraded articular cartilage, prolargin was among the top 16 proteins from which the most abundantly released peptides originated from^25^. MMPs are activated upon inflammation, which is the first step in PsA tissue injury process. Cartilage degradation in PsA has been linked to upregulation of pro-inflammatory cytokines TNF and IL-17—which, in turn, can lead to an increased production of MMPs^26–28^. Indeed, MMP levels have been reported elevated in synovial fluid and serum of patients with PsA^29,30^.

Thus, the aim of this study was to develop, validate and characterize a novel biomarker PROM targeting a MMP-generated neo-epitope specific fragment of prolargin, and thereafter evaluate its abilities as a biomarker for PsA. The neo-epitope biomarker technology is based on targeting the amino acid sequence at the new terminal of generated fragments.

## Results

### Production and characterization of monoclonal antibodies

Antibody-producing clones were generated after fusion between mouse spleen cells and myeloma cells and the monoclonal antibody with the best native reactivity, peptide affinity, and stability for the assay was identified. Based on reactivity, we selected antibody clone NBH228-1F7-1D9-1D6-2D12. Antibody isotype was determined to be IgG1, kappa.

### Technical evaluation

During assay development we optimized the following parameters: buffers (pH and salt content), incubation temperature and time, coater and antibody ratio, testing of labeled and unlabeled antibody, stability and specificity of the mAb. We have selected the coater:mAb ratio based on B/B0 checkerboard assay results (Supplementary Fig. S1) together with considering the total signal strength and serum sample screening results (criteria: healthy human serum samples should be on the measurement range). PBS-based buffer was selected due to a better pH long-term stability.

The technical validation was performed to evaluate the newly developed PROM assay. A summary of all the technical tests, can be found in Table 1. The assay measurement range lower limit (LLMR) and upper limit (ULMR) were determined to be 0.08 ng/ml and 2.04 ng/ml, respectively. The mean intra- and inter-assay variation based on 10 independent runs yielded a 4.1% and 10.6% recovery, respectively. Linearity of human serum was assessed from undiluted to an eightfold dilution. The analyte stability was acceptable for both 2–5 times freeze/thaw cycles and prolonged storage of human serum samples. Spiking of standard peptide in human serum resulted in a mean recovery of 63%, while serum in serum spiking resulted in a mean recovery of 93%. Neither low nor high levels of biotin, hemoglobin and lipids interfered with the levels of PROM in human serum.

**Table 1:** PROM ELISA technical validation data.

Technical validation	PROM
IC50	0.43 ng/mL
Detection range	0.08–2.04 ng/ml
Intra-assay variation^a^	4.1 (1.18–6.86)
Inter-assay variation^a^	10.6 (4.85–17.81)
Dilution (1:2) recovery in serum^a^	100 (83–94)
Dilution (1:2) recovery in heparin plasma^a^	101 (88–122)
Interference biotin, low/high	≤ 107%/ ≤ 102%
Interference lipemia, low/high	≤ 103%/ ≤ 102%
Interference hemoglobin, low/high	≤ 112%/ ≤ 107%
Freeze–thaw analyte stability (2 cycles)^a^	105 (102.2–108.6)
Analyte stability (stress test)	At 4 °C, the mean percent (SD) recovery was: 100 (5.8), 107 (14.1), 108 (20.0), 109 (3.8) after 2, 4, 24, and 48 h, respectively At 20 °C: 96 (10.9), 99 (17.0), 115 (11.0), 113 (14.4) after 2, 4, 24 and 48 h, respectively
Antibody stability (stress test)	Mean recovery (SD) after 7 days incubation: At 4 °C: 114% (15.6) At 20 °C: 110% (25.8)
Spike-in recovery (serum in serum)^a^	93 (80–115)

^a^Mean (range) recovery percentages are reported.

**Figure 1:**
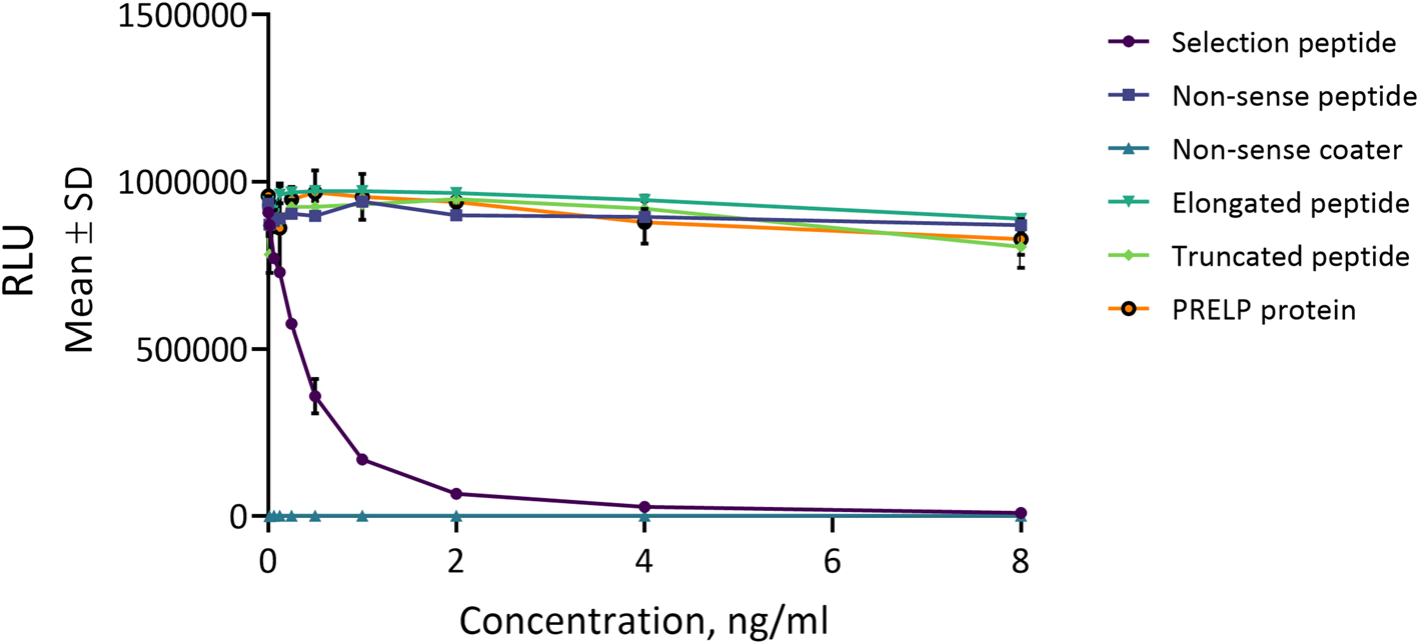
Specificity of the PROM assay. The assay is specific to its selection peptide (HDFSSDLENV) and does not recognize truncated (DFSSDLENV), elongated (FHDFSSDLENV), or recombinant human prolargin protein (Gln21-Ile382), nor non-sense peptides (KSVDQASSRK). A twofold dilution of the peptides were added starting from 8 ng/ml. The background signal was tested using a non-sense coating peptide (Biotin-KSVDQASSRK). The data is presented as relative light units (RLU) function of peptide concentration.

**Figure 2:**
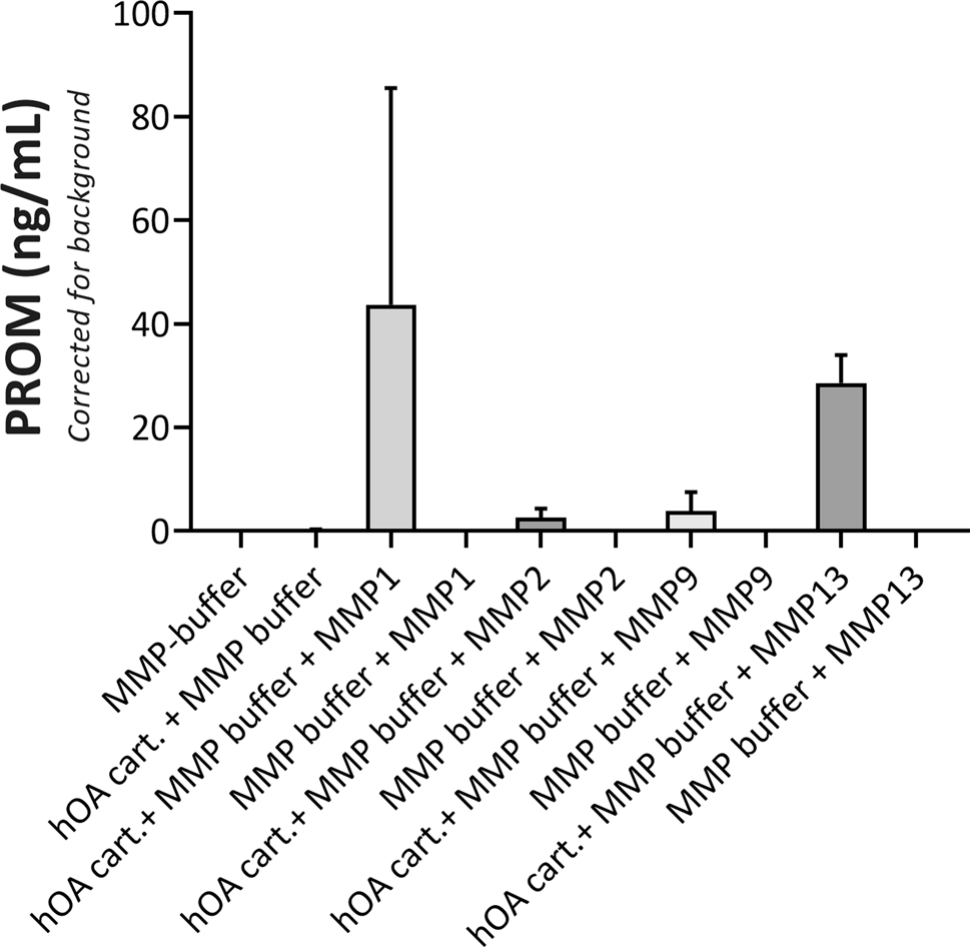
PROM release from MMP-cleaved human articular cartilage. PROM fragment is released from MMP-1, MMP-2, MMP-9 and MMP-13-cleaved human articular cartilage (hOA cart.) obtained from end-stage OA patients undergoing total joint replacement surgery. Data shown is from one (n = 1) representative cartilage sample. Error bars represent standard deviation for two biopsies from the same cartilage sample.

The PROM assay specificity was tested towards synthetic peptides, to confirm the specificity of the cleavage fragment. No reactivity was found towards the elongated peptide, truncated peptide, nonsense peptide and nonsense coater (Fig. 1). In vitro cleavages experiments showed the assay PROM was generated predominantly by collagenases MMP-1 and MMP-13, and to a lesser extent by gelatinases MMP-2 and MMP-9 (Fig. 2).

### Biological evaluation

**Figure 3:**
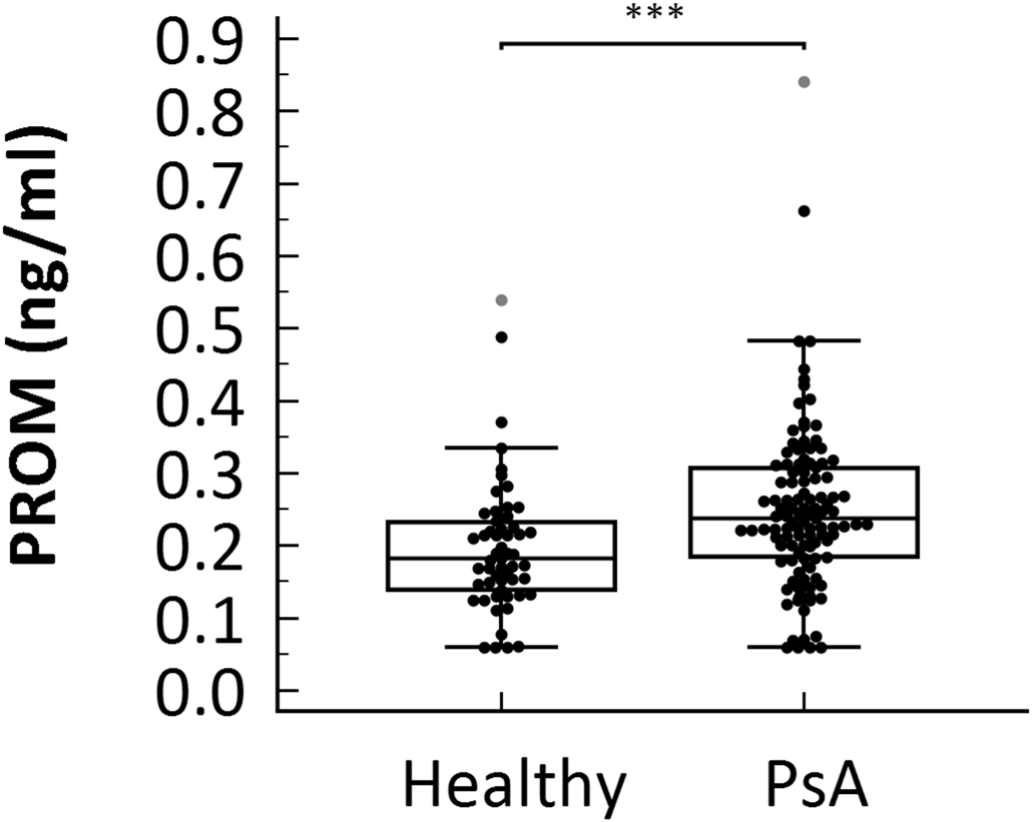
Results from the biological relevance validation cohort. Serum levels of PROM was assessed in healthy controls (n = 55) and patients diagnosed with PsA (at baseline, n = 111). Data was analyzed using a Mann–Whitney U test. Data are presented as Tukey box-and-whisker plot. Significance threshold was set at p < 0.05, ***p = 0.0003.

**Figure 4:**
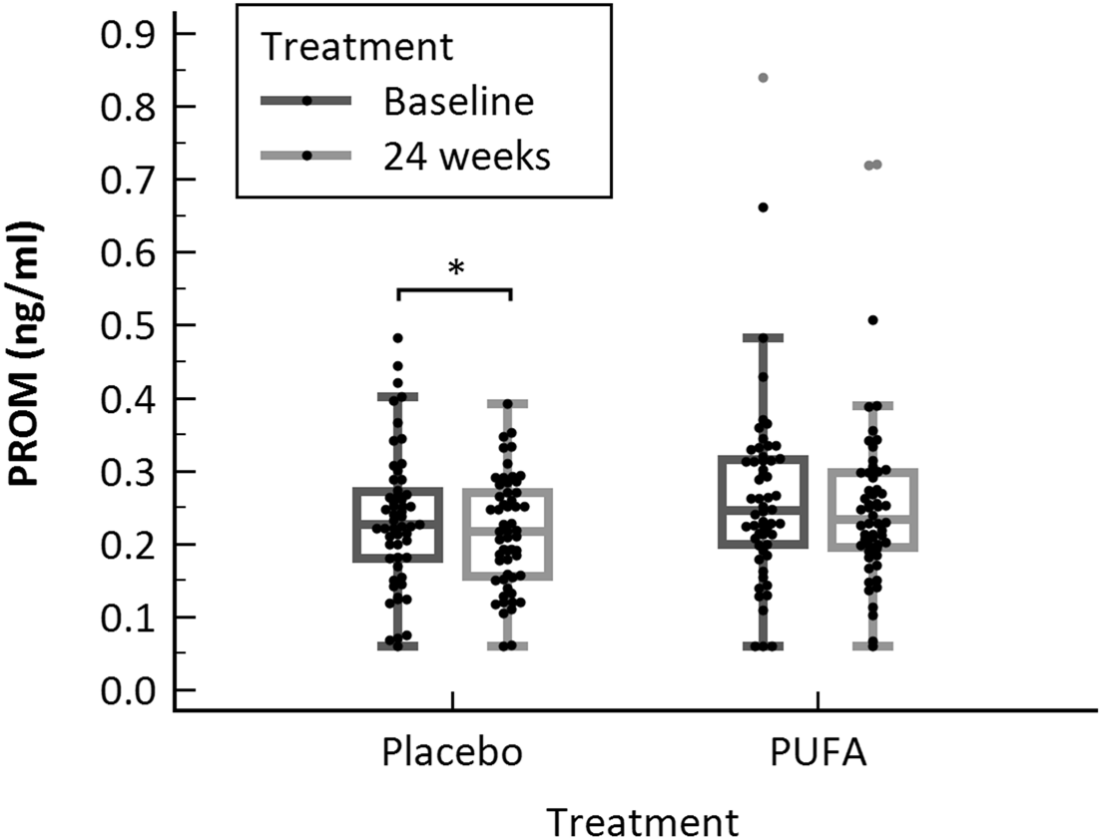
PROM levels in placebo and n-3 PUFA treated patients at baseline and 24 weeks. PROM levels were decreased in the Placebo group after 24 weeks. Significance threshold was set at p < 0.05 (Wilcoxon’s paired signed-rank test) and data is presented as Tukey box-and-whisker plot. *p = 0.049.

**Figure 5:**
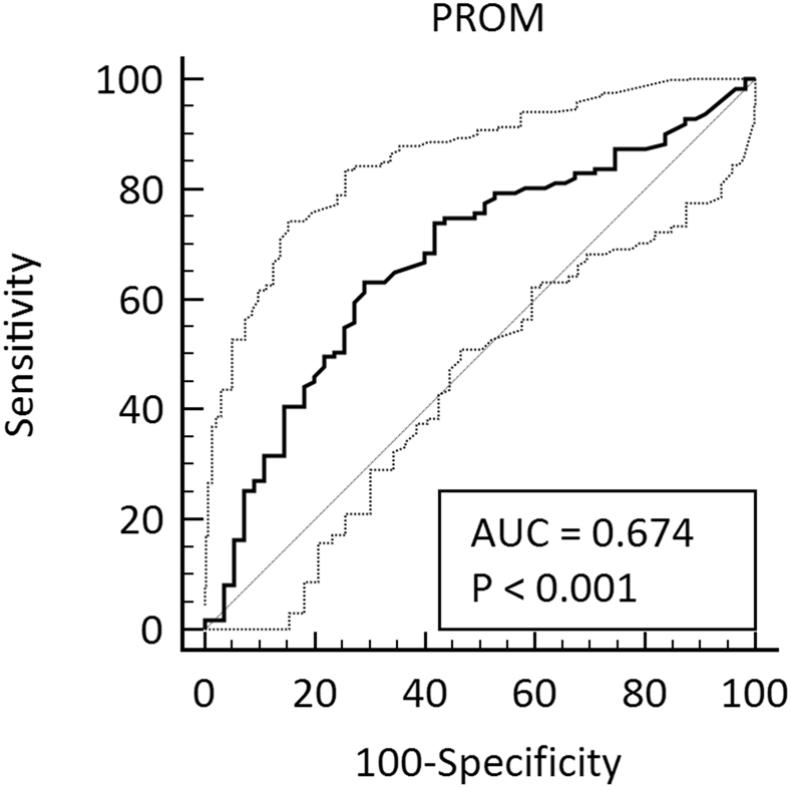
ROC curve analysis of the PROM biomarker for distinguishing subjects with PsA from healthy controls. AUC = 0.674, 95% CI 0.597–0.744, P < 0.001, Youden index J = 0.34, sensitivity 63.1, specificity 70.9, criterion > 0.22 (calculated with MedCalc DeLong et al., 1988 method).

PROM was measured in a biological validation cohort (Table 2), which consisted of healthy controls and patients with PsA at baseline. All patients and healthy controls were Caucasian. PROM levels were lower (p = 0.0003) in healthy individuals (median: 0.18; IQR: 0.14–0.23) compared to patients with PsA (median: 0.24, IQR: 0.19–0.31) (Fig. 3). After 24 weeks, the level of prolargin showed a borderline significant decrease in the placebo group (p = 0.049), but no difference was observed in the n-3 polyunsaturated fatty acid (PUFA) treated group (Fig. 4). There was no significant difference between placebo and n-3 PUFA groups at baseline, nor at 24 weeks (Fig. 4). PROM levels also did not correlate with disease scores, sex, age, BMI or disease duration (Tables 3 and 4). The diagnostic power (AUROC) of PROM for separating a patient with PsA from healthy controls was 0.674 (95% CI 0.597–0.744, *P* = 0.001, Fig. 5).

**Table 2:** Patient demographics for the biological validation cohort.

Variable	Controls n = 55	Placebo n = 55	PUFA n = 56	p-value	Completeness of data, %
Age, years	51.1 (14.6)	51.0 (11.8)	53.9 (11.7)	0.413^a^	100
Sex, male	23 [41.8]	24 [43.6]	23 [41.1]	0.961	100
PsA duration, years	N/A	12.5 (6.0–18.0)	10.0 (5.5–19.0)	0.839^b^	99
BMI (kg/m^2^)	N/A	26.9 (24.2–31.9)	28.3 (24.6–31.8)	0.528^b^	100
SJC	N/A	0.0 (0.0–1.0)	0.0 (0.0–0.0)	0.310^b^	100
TJC	N/A	2.0 (0.0–6.0)	1.0 (0.0–5.5)	0.892^b^	100
ASDAS	N/A	2.4 (1.4–3.2)	2.0 (1.4–2.7)	0.146^b^	100
BASDAI	N/A	35.0 (15.5–57.0)	28.5 (14.5–55.5)	0.365^b^	100
BASMI	N/A	0.0 (0.0–0.0)	0.0 (0.0–0.0)	0.167^b^	100
DAPSA	N/A	12.3 (7.4–17.3)	9.3 (3.9–17.9)	0.184^b^	100
DAS-28	N/A	2.6 (2.0–3.4)	2.5 (1.9–3.2)	0.246^b^	100
LEI	N/A	1.0 (0.0–2.0)	1.0 (0.0–2.0)	0.983^b^	100
PASI	N/A	1.2 (0.3–2.7)	0.6 (0.0–3.5)	0.717^b^	100
SPARCC	N/A	2.0 (0.0–4.0)	1.0 (0.0–4.5)	0.744^b^	100
CRP (mg/L)	N/A	4.2 (2.8–8.5)	2.9 (2.4–6.1)	0.193^b^	100
VAS global (mm)	N/A	45.0 (21.0–62.5)	24.5 (11.5–56.0)	0.065^b^	100
VAS pain (mm)	N/A	41.0 (20.5–57.8)	25.5 (11.5–48.0)	0.063^b^	100
Arthritis on X-ray	N/A	28 [50.9]	24 [42.9]	0.398	100
NSAID use	N/A	30 [54.5]	36 [64.3]	0.298	100
DMARD use	N/A	37 [67.3]	47 [83.9]	0.042	100
Documented coronary heart disease	N/A	3 [5.5]	4 [7.1]	0.716	100
Hypertension	N/A	12 [21.8]	17 [30.9]	0.282	99
Hypercholesterolemia	N/A	6 [10.9]	17 [30.4]	0.012	100
Total cholesterol, mmol/l	N/A	4.8 (4.1–5.3)	4.9 (4.4–5.3)	0.394^b^	100
Systolic BP, mmHg	N/A	134.5 (119.0–143.0)	139.0 (120.3–151.0)	0.281^b^	98
Diastolic BP, mmHg	N/A	80.5 (74.0–89.0)	82.0 (73.0–87.8)	0.998^b^	98

Categorical variables are expressed as number n [%] and were compared using Chi-square test; continuous variables as mean (SD) or median (IQR).

*SD* standard deviation, *BMI* body mass index, *SJC* swollen joint count, *TJC* tender joint count, *ASDAS* the ankylosing spondylitis disease activity score, *BASDAI* bath ankylosing spondylitis disease activity index, *BASMI* bath ankylosing spondylitis meterology index, *DAPSA* disease activity in psoriatic arthritis score, *DAS-28* disease activity score-28 joints, *LEI* leeds enthesitis index, *PASI* psoriasis area and severity index, *SPARCC* spondyloarthritis research consortium of Canada, *CRP* C-reactive protein, *VAS* visual analogue scale, *NSAID* non-steroidal anti-inflammatory drug, *DMARD* disease modifying anti-rheumatic drugs.

^a^ANOVA.

^b^Mann–Whitney U-test.

**Table 3:** Association with clinical assessment at baseline (all PsA patients pooled): Spearman’s correlations between baseline PROM biomarker concentration and other parameters for disease activity in the population with PsA.

	Spearman’s rho	p
ASDAS	0.016	0.864
BASDAI	0.018	0.855
BASMI	0.043	0.651
CRP	0.000	0.999
DAPSA	0.072	0.451
DAS-28	0.090	0.346
HAQ	− 0.049	0.611
LEI	0.156	0.101
PASI	0.046	0.633
SJC	0.064	0.501
SPARCC	0.110	0.249
TJC	0.121	0.207
VAS global	− 0.012	0.904
VAS pain	0.001	0.994
Arthritis on x-ray	0.063	0.514

*ASDAS* the ankylosing spondylitis disease activity score, *BASDAI* bath ankylosing spondylitis disease activity index, *BASMI* bath ankylosing spondylitis meterology index, *CRP* C-reactive protein, *DAPSA* disease activity in psoriatic arthritis score, *DAS-28* disease activity score-28 joints, *HAQ* health assessment questionnaire, *LEI* leeds enthesitis index, *PASI* psoriasis area and severity index, *SJC* swollen joint count, *SPARCC* spondyloarthritis research consortium of Canada, *TJC* tender joint count, *VAS* Visual analogue scale.

**Table 4:** Association between PROM and sex, age, BMI, or disease duration.

	Age	Sex	Years since diagnosis	BMI
**PROM at baseline**				
rho	0.103	− 0.013	− 0.027	− 0.167
P	0.2801	0.8962	0.7759	0.0791

*BMI* body mass index.

## Discussion

PsA is a progressive and disabling disease that remains underdiagnosed^6^. Although Classification Criteria for Psoriatic Arthritis (CASPAR criteria)^31^ are used for providing guidance to the clinicians and for enrolling patients in clinical trials, we need biomarkers to better account for the diverse clinical presentation of PsA and to facilitate personalized medicine. Furthermore, the ability to detect patients with poor prognosis would help to choose which patients need a more aggressive treatment, and the ability to identify which medications would work best for an individual patient with PsA. This would address the fact that many patients must cycle through a number of medications before finding the one that works for them^8,32^.

In this study we developed, validated and characterized a novel competitive ELISA detecting a MMP-generated prolargin fragment (PROM) using a monoclonal antibody. The main findings in this study were: (1) The novel assay was technically robust and specific to the target sequence, (2) the fragment was present in serum and plasma, (3) PROM was upregulated in patients with PsA compared to healthy controls, and (4) PROM did not show a difference in patients treated with PUFA treatment. We investigated the PUFA treatment, as marine n-3 PUFAs have previously reduced inflammation, joint pain, and NSAID use in patients with rheumatoid arthritis (RA), and n-3 PUFA supplementation in patients with PsA led to a reduced use of NSAIDs and paracetamol^33^ as well as an improved heart rate and heart rate variability^34^. However, there was no correlation to the disease scores, which we also do not see with PROM. Although it was not discriminatory in a diagnostic way, PROM might be useful combined with another biomarker or feature.

The PROM ELISA was characterized as being a technically robust assay—it recognized only the selection peptide in specificity assessment, and showed dilution recovery, stability and interference tests within the accepted range ± 20%. Additionally, the inter- and intra-assay variation was acceptable with values of 4.1% and 10.6%, respectively. The analyte and antibody were stable at 20 °C for up to 48 h and 7 days at 37 °C, respectively.

The work reported here has limitations—the healthy patients, despite being matched on age, race and sex, did not come from the same source as the PsA patients. Furthermore, the patients included in the PsA cohort had low disease activity /remission with approximately 75% of the participants in treatment with DMARDs. Olive oil was used as control, and while olive oil has often been used for this purpose in studies investigating the effects of n-3 PUFA, it may itself have anti-inflammatory actions. Additionally, we did not test if the biomarker is PsA-exclusive, nor did we look into cell signaling. In RA, cartilage tissue is destroyed and prolargin fragments are released into synovial fluid, where it is hypothesized that they interact with complement system components and downregulate complement activation, which has been shown to play a role in both initiating the inflammatory state and in maintaining the inflammation during chronic disease^35^. Therefore, in future studies it would be interesting to investigate whether the neoepitope affects the complement system. Additionally, now, most biomarkers are not PsA specific, but rather indicate synovial inflammation and/or cartilage turnover in inflammatory joint disease. For example, active serum MMP3 levels decreased in both ankylosing spondylitis (AS) and RA after anti-TNFα treatment^36^. Therefore, in the future studies PROM should be measured in other inflammatory joint diseases to ascertain whether it is PsA-specific, and whether it can distinguish patients with psoriasis that do not have PsA. The assay should also be measured in studies for treatment efficacy (e.g. IL-17/IL-23 inhibitor studies) for further PROM validation in PsA.

In summary, MMP-cleaved prolargin can be quantified in serum by the PROM assay and has the potential to separate patients with PsA from healthy controls. To our knowledge this is the first study to develop a specific neo-epitope ELISA biomarker of prolargin and measure it in patients diagnosed with PsA.

## Materials and methods

### Reagents

Synthetic peptides were purchased from Genscript (Piscataway, NJ, US) and chemicals were purchased from Sigma-Aldrich (St.Louis, MO, US) or Merck (Whitehouse Station, NJ, US).

### Production of monoclonal antibodies

Antibodies were raised against the 10 amino acid sequence 340′.HDFSSDLENV’349 from prolargin cleaved in the C-terminus, which was discovered by mass spectrometry analysis of MMP-cleaved human articular cartilage samples and verified by BLAST to be unique for prolargin using NPS@: Network Protein Sequence Analysis with the UniprotKB/Swiss-prot database^25,37^. Five mice were immunized by subcutaneous injection of 200 µL emulsified antigen and 50 µg immunogenic peptide (HDFSSDLENV-GGC-KLH) in 6–7 weeks old Balb/C female mice using Stimune Immunogenic Adjuvant (SPECOL) (Invitrogen, Carlsbad, CA, US). This was repeated every 2nd week until stable serum antibody titer levels were reached. The mouse with the highest serum titer was selected for fusion and rested for a month. Then, the mouse was boosted intravenously with 50 µg immunogenic peptide in 100 µL 0.9% NaCl solution three days before isolation of the spleen for cell fusion. The mouse spleen cells were fused with SP2/0 myeloma cells as described by Gefter et al. (1977), to produce hybridoma cells^38^. After this, the clones were plated into 96-well microtiter plates for further growth and the limiting dilution method was applied to promote monoclonal growth. The positive clones were picked out for preliminary characterization including checkerboard and peptide inhibition. Two clones showed peptide inhibition and were chosen for further growth. A competitive ELISA was performed on streptavidin-coated plates to screen supernatant reactivity. HDFSSDLENVK-Biotin was used as screening peptide, while the selection peptide HDFSSDLENV was used to test specificity of the clones. Antibody isotype determination was performed using commercial Rapid ELISA Mouse mAb Isotyping Kit (Invitrogen, Carlsbad, CA, US) following the manufacturer’s instructions. Clone 1F7 was selected for assay development based on specificity, selectivity and titer. Supernatant was collected from the hybridoma cells and immunoglobulins purified using HiTrap Protein G HP affinity columns according to manufacturer’s instructions (GE Healthcare Life Science, Buckinghamshire, UK).

### PROM ELISA methodology

The PROM ELISA was as follows: 96-well streptavidin-coated ELISA plates (cat. no. 655995, Greiner Bio-One, Austria) were coated with 2.5 ng/mL biotinylated peptide dissolved in assay buffer (10 mM PBS-BTB, 8 g. NaCl, pH 7.4), 100 µL/well and incubated for 30 min at 20 °C in the dark with 300 rpm shaking. Plates were washed five times in washing buffer (20 mM TRIS, 50 mM NaCl, pH 7.2). Subsequently, 20 µL of selection peptide or sample were added to appropriate wells, followed by 100 µL of 75 ng/mL horseradish peroxidase (HRP) conjugated monoclonal antibody. The plates were incubated for 20 h at 4 °C with shaking, and subsequently washed in washing buffer. Hundred µL per well of BM Chemiluminescence ELISA Substrate (POD) (cat. no. 11582950001, Roche, Switzerland) working solution was then added to the plate and incubated for 3 min at 20 °C with shaking. The plate was analyzed by a SpectraMax M5 reader (Molecular Devices, CA, USA) with settings: luminescence, Lm1 = 440 nm, Lm2 = 650 nm. A standard curve was generated by serial dilution of the selection peptide and plotted using a 4-parametric mathematical fit model. Standard concentrations were 8, 4, 2, 1, 0.5, 0.25, 0.125, 0.063, 0.031, 0.016 and 0 ng/ml. Each plate included five kit controls to monitor inter-assay variation. All samples were measured within the measurement range of the assay.

### Technical evaluation

To assess the linearity of the assay, four healthy human serum samples were used. The linearity was calculated as a percentage of recovery of the undiluted sample. The intra- and inter-assay variations were determined by ten independent runs of eight quality controls (QC) and two kit controls run in double determinations. Each run consisted of two replicas of double determinations of the samples. Lower limit of measurement range (LLMR) and upper limit of measurement range (ULMR) was calculated based on the 10 individual standard curves from the intra- and inter-assay variation. The analyte stability was determined for three healthy human serum samples which were incubated at either 4 °C or 20 °C for 2, 4, 24 and 72 h respectively. The stability of the samples was evaluated by calculating the percentage variation in proportion to the sample kept at − 20 °C (0 h sample). Stability of the analyte was also determined for three healthy human serum samples, exposed to four freeze and thaw cycles. The percentage of recovery was calculated using a reference sample that underwent only one freeze/thaw cycle. Stability and specificity of the generated monoclonal antibody to the standard peptide was also studied. The stability study included incubation of primary antibody at 4 °C, 20 °C and 37 °C for 24 h, 72 h and 7 days. The specificity study included a nonsense peptide (KSVDQASSRK), an elongated peptide (FHDFSSDLENV), a truncated peptide (DFSSDLENV), a CHO-derived human PRELP protein (Gln21-Ile382, R&D Systems, MN, USA) and a nonsense coater (Biotin-KSVDQASSRK), used for determination of cross-reactivity. Accuracy was measured in three healthy human serum samples. The samples were spiked with known concentrations of a serum sample and spiking recovery was determined by calculating the percentage recovery of serum spiked in. Interference was measured in healthy human serum spiked with biotin (low = 3 ng/ml, high = 9 ng/ml), hemoglobin (low = 2.5 mg/mL, high = 5.0 mg/mL), or lipids (low = 1.5 mg/mL, high = 5.0 mg/mL). The interference was calculated as the percentage recovery using the analyte in non-spiked serum as reference. To define the standard concentration of PROM, the normal range was determined by analyzing serum panel from 32 healthy donors (Supplementary Table S1) purchased from BioIVT (NY, USA).

### Cleavage analysis

To evaluate the ability of MMP-1 (cat. no. G04MP01C, Giotto Biotech, Italy), MMP-2 (cat. no. G04MP02C, Giotto Biotech, Italy), MMP-9 (cat. no. G04MP09C, Giotto Biotech, Italy), and MMP-13 (cat. no. GAD00317, Giotto Biotech, Italy) to generate the PROM neo-epitope, 60 mg of human articular cartilage was incubated for 72 h at 37 °C with the aforementioned proteases. Six conditions were prepared as controls containing either MMP cleavage buffer, cartilage in cleavage buffer without MMPs, and individual MMPs in cleavage buffer without cartilage. The cleavage reactions were stopped using 1:1,000 dilution from 5 mM ethylenediaminetetraacetic acid (EDTA). Cleavage products were stored at − 20 °C until analysis. All samples were prepared in duplicates.

### Biological validation of PROM

The PsA cohort is described in detail in the study by Kristensen et al. (2016). Briefly, the study was conducted as a randomized, double-blind, placebo-controlled trial, where patients with PsA (defined by CASPAR) were supplemented with 3 g of n-3 PUFA or olive oil (control) daily for 24 weeks. Inclusion criteria were adults above 18 years of age with any disease activity, and exclusion criteria were: documented known cardiac arrhythmias, treatment with biological drugs, or treatment with oral corticosteroids. Compliance was assessed by counting capsules during the last visit and patients missing > 15% of capsules were excluded from analysis. Clinical assessment and blood samples (in a non-fasting state) were obtained at baseline and after 24 weeks of follow-up. Samples were processed immediately and stored at − 80 °C until analysis^33,34^.

For comparison, a panel of age-, sex- and race- matched healthy controls was obtained from two commercial vendors: Discovery Life Science (AL, USA) and Lee Biosolutions (CA, USA). The healthy controls were collected in 2018 after informed consent and approval by the local Ethics Committee and with compliance with the Helsinki Declaration of 1975, processed immediately after collection according to standard operating procedures and stored at − 80 °C until analysis.

All patients from the PsA cohort gave their written informed consent and the regional ethics committee of North Denmark region approved the study (reference number N20120076). The study was conducted in accordance with the Declaration of Helsinki and registered at ClinicalTrials.gov (NCT01818804). Good clinical practice (GCP) inspectors monitored the study and the GCP ethical and scientific quality requirements were followed^33,34^.

### Statistical analysis

Characteristics of the cohort are presented as a number (frequency) and percentage for categorical variables and mean (standard deviation) or median (interquartile range) for normally and non-normally distributed continuous variables, respectively. Statistical differences for categorical variables were assessed using a Chi-squared test. For numerical variables, parametric tests (ANOVA, t-test) were used for normally distributed data, and nonparametric (Mann–Whitney U test, Kruskal–Wallis test and Spearman correlation) for non-normally distributed data. For all statistical analysis performed, a p-value below 0.05 was considered significant. Statistical analysis and graphs were performed using MedCalc Statistical Software version 18.11.6 (MedCalc Software bvba, Ostend, Belgium) or GraphPad Prism version 8 (GraphPad Software, Inc., CA, USA). PROM levels below the LLMR were given the LLMR value.

### Ethical statement

The production of monoclonal antibodies performed in mice was approved by the Danish National Authority (The Animal Experiments Inspectorate) under approval number 2013-15-2934-00956. All animals were treated according to the guidelines for animal welfare.

## Supplementary information

Supplementary file 1

## Data Availability

The datasets generated during and/or analyzed during the current study are available from the corresponding author on reasonable request.
