# Supplementary material for: A novel biomarker of MMP-cleaved prolargin is elevated in patients with psoriatic arthritis
Source: Sci Rep. 2020 Aug 11;10:13541. doi: 10.1038/s41598-020-70327-0 (PMC7419545; doi:10.1038/s41598-020-70327-0)
Supplement: Supplementary file 1 [file 41598_2020_70327_MOESM1_ESM.docx]

# Title page

**Title:** A novel biomarker of MMP-cleaved prolargin is elevated in patients with psoriatic arthritis

**Authors:** Dovile Sinkeviciute^1,2^ *, Solveig Skovlund Groen^1,3^, Shu Sun^1^, Tina Manon-Jensen^1^, Anders Aspberg^2^, Patrik Önnerfjord^2^, Anne-Christine Bay-Jensen^1^, Salome Kristensen^4^, Signe Holm Nielsen^1,5^

**Affiliations:**

^1^Nordic Bioscience, Biomarkers and Research, Herlev, Denmark

^2^Department of Clinical Sciences Lund, Lund University, Lund, Sweden

^3^ Department of Biomedical Sciences, University of Copenhagen, Copenhagen, Denmark

^4^Department of Rheumatology, Aalborg University Hospital, Aalborg, Denmark

^5^Department of Biotechnology and Biomedicine, Technical University of Denmark, Kgs. Lyngby, Denmark

***Corresponding author:**

Dovile Sinkeviciute

Nordic Bioscience, Herlev Hovedgade 205-207, 2730 Herlev, Denmark & Lund University, 223 62 Lund, Sweden.

Telephone: +45 44 52 52 52, Fax: +45 44 52 52 51, E-mail: [dsi@nordicbio.com](mailto:dsi@nordicbio.com)


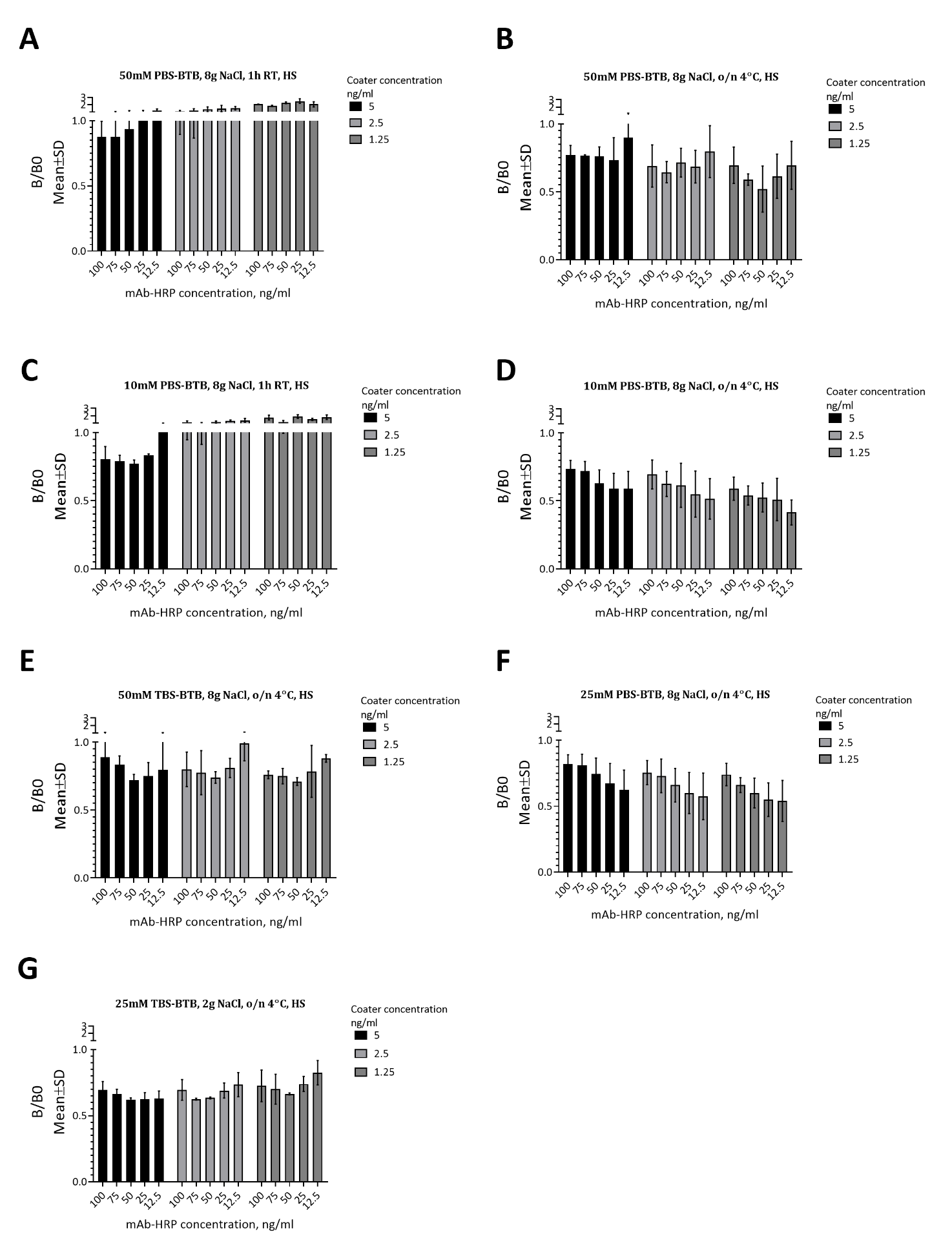


**Supplementary Figure S1.** **Assay settings optimization during assay development**: buffers (pH and salt content), incubation temperature (RT – room temperature) and time, coater and antibody ratio for measuring in healthy human donor serum (HS).
